# Competency for Japanese novice medical laboratory scientists: a Delphi method

**DOI:** 10.1186/s12909-022-03878-7

**Published:** 2022-12-16

**Authors:** Kiriko Maekawa, Sayaka Kotera, Hiroyuki Ohsaki

**Affiliations:** 1 Department of Medical Science, Divisin of Medical Education, Graduate School of Medicine, Kobe University, 7-5-1 Kusunokicho, Chuo, Kobe, Hyogo 650-0017 Japan; 2 Department of Medical Biophysics, Graduate School of Health Sciences, Kobe University, 7-10-2 Tomogaoka, Suma, Kobe, Hyogo 654-0142 Japan; 3 Department of Public Health, Graduate School of Health Sciences, Kobe University, 7-10-2 Tomogaoka, Suma, Kobe, Hyogo 654-0142 Japan

**Keywords:** Health professions education, Medical laboratory scientist, Competency, Novice, Delphi method, Japan

## Abstract

**Background:**

Competency is used to channel abilities into successful processes and is employed in the medical field. Globally, several laboratory competencies exist, but the job descriptions of Japanese medical laboratory scientists differ from those of other countries and little evidence-based information on novice medical laboratory scientist competency is available in Japan. This study clarified the competencies of novice medical laboratory scientists based on various expert opinions in Japan.

**Methods:**

The Delphi method was used to achieve an expert consensus on novice medical laboratory scientist competencies. We asked the participants to evaluate the importance of each item using the Likert scale and set 70% as the final consensus rate.

**Results:**

We obtained 106/400 (26.5%) and 95/106 (89.6%) responses from participants in rounds 1 and 2, respectively. Their professional experience mean ± standard deviation was 32.4 ± 6.0 years (range: 13–41). The average of each category consensus rate was > 99.1%. Ninety-five expert opinions converged and agreed that the competency comprised 8 categories and 54 items.

**Conclusions:**

The survey results revealed that novice medical laboratory scientists were expected to have relatively higher main laboratory skill competencies in the ‘Preparation and analysis’ category than in other categories. Nevertheless, competencies in other categories required basic skills. In addition, our competencies contained unique competencies compared with others due to their divergent roles and their environment. Further research is warranted to explore assessment tools by developing a competency scale, thereby helping clarify the differences between ability and correlated factors. The unique competencies scale can help assess the efficacy of educational programmes for Japanese medical laboratory scientists.

**Supplementary Information:**

The online version contains supplementary material available at 10.1186/s12909-022-03878-7.

## Background

Recently, the quality of medical care in Japan has been changing because of the declining birth rate and ageing population, thereby developing the medical environment and increasing the sophistication and specialisation of medical technology. These changes affect the work of doctors and nurses and several medical professionals, including a medical laboratory scientist (MLS). An MLS, also known as a clinical laboratory scientist, biomedical laboratory scientist and medical technologist, works in a medical laboratory. Specifically, in 2020, the spread of coronavirus disease 2019 (COVID-19) has led to an increased focus on polymerase chain reaction (PCR) and antibody testing, increasing the need for MLS [1]. To cope with these changes, training institutions for MLS have reviewed their educational content [2]. Recently, the Japanese Ministry of Health, Labour and Welfare (MHLW) has reviewed the school course content and the number of credits in the curriculum of training institutes for MLSs [3]; there has also been a move for aligning educational standards. Furthermore, the trends in MLS education have focused on the standardisation of educational content and clinical practice. However, there remains a lack of a clear standardisation of education for novice MLSs who have obtained national qualifications and are employed in various medical institutions [4]. The time available for in-service training in the dynamic medical field is limited, and training mid-career and senior MLSs who can supervise in line with the abilities of each novice is difficult.

The United States government office of personnel management defines competency as ‘a measurable pattern of knowledge, skills, abilities, behaviours and other characteristics that an individual needs to perform work roles or occupational functions successfully’ [5]. Competency is also used to manage the abilities into successful processes and is widely employed in human resource development [6]. The International Federation of Biomedical Laboratory Science (IFBLS) and the Centers for Disease Control and Prevention (CDC) have developed competencies for biomedical laboratory scientists and public health laboratory professionals [7, 8]. Moreover, seven pathology industry associations in Australia have developed competency-based standards for medical scientists working in diagnostic pathology settings [9]. The standards are the entry level of a scientist into this profession, reflecting a combination of qualifications, skills and the assumption of personal responsibilities and accountability [7]. The American Society of Clinical Pathologists and the Canadian Society for Medical Laboratory Science offer competency-based certification examinations [10, 11] that are useful in determining whether candidates have the skills and knowledge required to succeed as MLSs [12]. Thus, competency is regarded as essential to ensure the abilities of MLSs. Nonetheless, we could not find any studies related to Japanese novice MLS competencies on PubMed, published from the earliest date up to October 2019. Because the importance of competencies has been examined and utilised in work and examinations internationally, the development of competencies in Japan would effectively improve MLS quality.

The worldwide movement towards having medical laboratories accredited by the International Organisation for Standardisation (ISO) 15189 is another reason for enhancing competency [13]. This international standard specifies requirements for quality and competence in a medical laboratory [14] and partly relates to services for clinical physiology. Hence, competency is clearly essential and fundamental for MLSs.

This study aimed to identify the competencies of novice MLSs using the Delphi method. The Delphi method is an interactive process for collecting and distilling the anonymous judgements of experts using a series of data collection and analysis techniques interspersed with feedback [15], and the approach enhances reliability [16]. There is little evidence-based information about the novice competencies of MLSs in Japan, and it would be essential to gather various expert views. These competencies are the minimum requirement for MLSs to be licenced and perform their jobs and have the advantage of setting clear goals for them and providing their supervisors with a systematic and objective way of educating them. Additionally, our study is expected to be used for their primary education in training schools.

## Methods

### Study design

This study used the Delphi method [17–19], which is broadly employed in medical and health fields [20–22], a useful tool that looks for answers to normative questions in the education area [23] to arrive at an expert consensus on the competencies of novice MLSs. The Delphi method is flexible [24], and we utilised the modified Delphi method [25], generating a draft competency using a literature review and some expert interviews rather than an initial round of the Delphi method. Subsequently, we distributed the competency questionnaires to gather the opinions of experts in Japan by using the Delphi method. Our expertise focused on novice MLSs, which were defined as those with fewer than 3 years of post-graduation employment experience [26]. In other medical professions, such as nursing, a competent nurse is regarded as one who has been on the job for 2–3 years [27]. Furthermore, in line with the threshold MLS experience of more than 10 years, a novice MLS generally refers to one with approximately 3 years of experience after graduation. Therefore, we used the threshold of fewer than 3 years of after-graduation experience to define a novice MLS.

### Instrument development

We developed a draft questionnaire by reviewing overseas literature pieces and publications on competencies [7, 8, 13]. First, a draft questionnaire was constructed and modified based on the IFBLS’s core competence [7] subjects for biomedical laboratory scientists working in a general laboratory setting. Some items were modified for appropriateness with the clinical laboratory setting and the Japanese context. Second, it was compared with the competency of the CDC guidelines and the association of a public health laboratory [8] to confirm any significant differences in terms of concept or direction. Lastly, some competency was added n referring to ISO 15189 [13] and other related literature about Japanese MLS requirements. The final modification was conducted downward from the professional to novice level. The content and face validity of the draft were assessed by professors of MLS education and mid-career MLSs who were not novices but were still non-experts. Through all processes, the draft questionnaire was approved to become the first round Delphi questionnaire.

### The questionnaire

In the questionnaire, we asked the participants to evaluate the importance of each competence using a 4-point Likert scale (4 = important, 3 = somewhat important, 2 = somewhat unimportant and 1 = unimportant). Japanese were likely to select midpoints in a questionnaire scale [28], and lower points would reduce the burden on participants in clinical settings. Therefore, we chose a 4-point Likert scale for our study. Futhermore, our study defined consensus as a positive agreement if the participants chose either 3 or 4 and no consensus as a negative agreement if they chose either 1 or 2 [25]. The recommended consensus rate of the Delphi method ranges from 51 to 70%, with 70% indicating more cautious [17]. Our study set 70% as the final consensus rate for each item, with two criteria [24]. An item receiving 4 points by more than 70% participants was automatically approved unless the item obtained comments and suggestions from the participants. Conversely, if an item received 1 point by more than 70% participants, it was automatically rejected. The item, except for the two criteria, would be approached on the basis of the results and comments of the previous questionnaire round. Based on the two criteria, the Delphi survey was planning to conduct three rounds of administering the questionnaire. In the first round, we also asked about the adequacy of the first questionnaire and any comments for our study as well as about ideas regarding any other necessary competency items with an open-ended question. The study was undertaken at the beginning of the COVID-19 pandemic from January to March 2020.

### Participants

The participants comprised experienced MLSs who worked at hospitals in Japan. Generally, a hospital has one head of MLSs, and they manage their laboratory and have enough knowledge, techniques, coaching skills and MLS experiences. Morover they takes reasonability for training and educating novice MLSs. As expert MLSs, the study set the following criteria: (1) held the national qualification for MLSs in Japan, (2) work experience as an MLS for more than 10 years and (3) heads of MLSs or equivalent in a clinical setting. Further, the hospital itself should be registered with the MLHW and have more than 300 beds [29]. In December 2020, 1,001 hospitals met the criteria through Japan. Final responses of at least 30 MLSs were targeted, which was recommended as the productive performance of the Delphi method [18, 20], and 400 hospitals were randomly chosen from 1,001 hospitals after stratified sampling, referring to the response rate reported in a previous study on Japanese MLSs [30]. The stratified sampling was used to reduce the sampling bias of regions and scales of hospitals.. The survey questionnaires were distributed by post all over Japan by using the postal address the MLHW described on the website [29]. Our research explanation form and questionnaire, which included a consent form, were sent to 400 hospitals during the first survey. Participants who provided their consent and responded in round 1 were included in round 2. To maintain anonymity, all postal addresses and names of the hospitals were managed by identification numbers and employed in a self-addressed envelope without any participant information.

### Data gathering

The participants were given the first questionnaire, comprising 8 categories and 51 competencies on a 4-point Likert scale. Two free comment boxes were added in respect of their right to free opinion. Regarding demographics, position, years of experience as an MLS and the number of beds and MLS in the participant hospital were queried. The second survey was administered to the first participants, and they received the second questionnaire analysed based on the first survey with descriptive statistics and the comment results summarised. It comprised 8 categories and 54 competencies on the same rate scale as well as reasons for ratings of 2 or 1.

### Group feedback

As group feedback, we enclosed the results of round 1 when we distributed the modified questionnaire of round 2. The results consisted of destructive statistics, participants' comments and the questionnaire-modification process. Moreover, we provided the results of round 2 and the final competency lists to all participants of our survey.

### Ethical consideration

Ethics approval to undertake this study was provided by the ethical committee of the Kobe University Graduate School of Health Sciences (approval number 904). Written informed consent was obtained from all participants, whose confidentiality was preserved.

## Results

### Demographics

Table 1 presents the response rates in the first and second rounds and the characteristics of the experts. A total of 106 responses were received from 400 participants (26.5%) in round 1 and 95 from 106 (89.6%) in round 2. In round 2, the questionnaire was distributed to all participants who provided valid answers in round 1. The experts’ years of experience mean ± SD was 32.4 ± 6.0 years (range: 13–41). The numbers of beds and MLSs they belong to were 499.8 ± 204.3 and 34.9 ± 22.1, respectively. Some participants reported that the number of beds was less than that of the MLHW registration.

**Table 1:** Total number and characteristics of the participants

	**Round 1**	**Round 2**
**Total**		
Collections/distributions	106/400	95/106
(Response rate)	(26.5%)	(89.6%)
**Characteristics**		
**Work experience years** n (%)		
10–19 years	4 (3.8)	
20–29 years	20 (19.0)	
≥ 30 years	81 (77.1)	
Mean ± SD (*n* = 105)	32.4 ± 6.0 (Range: 13–41 years)	
**Number of MLSs in their departments**		
Mean ± SD	34.9 ± 22.1 (Range: 5–122)	
**Number of beds in their departments**		
Mean ± SD (*n* = 103)	499.8 ± 204.3 (Range: 204–1200)	

All the collected items were analysed

The second survey continued the survey of the participants from the first survey

### Round 1

In the first round, the consensus rate in the Delphi survey was above 89.6% for 106 participants in all 51 items. Table 2 presents the overall consensus rate of each category. The Median has based on ratings on the level of importance by the participants.The reasons why item responses were answered as negative agreements were primarily related to the competency of difficulty for the novice MLSs. Comments on the adequacy of the questionnaire were all positive feedbacks and several comments held high expectations from our study. Further, comment responses suggested as new necessary competency items were indicated below. The ‘Preparation and analysis (general)’ category obtained comments about the competency of external quality assessment, while the ‘Preparation and analysis (physiology)’ category received comments about the competency of communication with patients. Moreover, ‘Medical safety management’ earned comments about the competency of a related disaster; ‘Interpersonal relationships’ obtained comments about the basic competency of reporting, informing and consulting about the task and ‘Medical ethics’ received comments about the competency of manner as a member of society.

**Table 2:** Overall novice MLS competency categories

Competency category	Round 1 (*n* = 106)			Round 2 (*n* = 95)		
	Items	Median	Consensus (%)	Items	Median	Consensus (%)
1. Sample collection	2	4	99.5	2	4	99.5
2. Preparation and analysis (general)	16	4	97.3	17	4	99.4
3. Preparation and analysis (specimens)	7	4	98.2	6	4	100.0
4. Preparation and analysis (physiology)	3	4	99.0	5	4	99.8
5. Medical safety management	6	4	96.9	6	4	99.1
6. Interpersonal relationships	6	4	96.4	6	4	99.5
7. Professional development	5	4	96.6	5	4	99.4
8. Ethics	6	4	98.1	7	4	99.4

In this round, there were primarily two patterns of the modifications of competency. One was a downward modification, which was adopted for items that had less than 70% rating under ‘important’ following the comments from the participants who were out of consensus. The other was adding new items and ideas to the first questionnaire following the comments from the participants who thought about necessary competencies other than the questionnaire items. Focusing on five items, which were median 3 in round 1, items 14 and 18 were in the ‘Preparation and analysis (general)’ category and item 29 was in the ‘Medical safety management’ category modified downwards per participants’ comments. Item 39 in the ‘Interpersonal relationships’ category was not suitable for medical workers in Japan and was modified as per the clinical setting expression. On the other hand, item 38 in the ‘Interpersonal relationships’ category was deleted to generate item 39 in the same category; those items were similar based on participants’ comments. Owing to the modification, the first questionnaire had 8 categories and 54 items. An additional file shows this in more detail [see Additional file 1].

### Round 2

In the second round, the consensus rate was above 97.9% for 95 participants in all 54 items, including the newly added items. The comments from the participants were almost the same as in the first round. As per the results, the questionnaire was partially modified and this round had become the final one because of the high consensus rate in each category (Table 2). Finally, the competency item had 8 categories and 54 items. An additional file shows this in more detail [see Additional file 2].

## Discussion

### Consensus rate

Our target for the final consensus rate, which was 70% and prudence, exceeded 97% in each category. The average consensus rate of each category was exceedingly high, and it was above 99.1%. The opinions of the 95 experts converged and they agreed on the competency in this study.

### Number of iterations

In this study, we modified the number of Delphi method iterations from three to two. The consensus rate of round 2 had already surpassed 97.9%, which was significantly higher than 70%, the final consensus rate we prudently set in the study. Previous studies revealed that two or three rounds were sufficient and most of the studies involved two rounds [31]. Therefore, we adjusted the number of iterations according to participants’ burden. After analysing round 2, we provided group feedback of round 2 to all respondents as the final research results.

### Findings

The following characteristics of competency were noted. First, ‘Sample collection’ and ‘Preparation and analysis (physiology)’ categories as well as 16 items were unique job competencies because Japanese MLSs do tasks that are unique to this country. This difference was due to the divergent roles of MLSs in Japan compared to other countries in Europe or the United States. For example, jobs like venous blood collection of patients and physiology examination are categorised under the MLS qualification in Japan but appointed to other professionals abroad [32]. The ‘Sample collection’ category was considered more critical, owing to the role expansion and increasing demand of PCR for COVID-19. The result clarified that the participants supported the importance of the categories. Additionally, the ‘Preparation and analysis (physiology)’ category included examination and communication skills with the patients. One of the MLS roles is creating a comfortable environment where patients can undergo a physiological examination based on the quality of life [33]. Competency included ‘examination of patients with safety and comfort’ in such categories, supporting the role of MLS.

Second, as per participant comments, ‘Medical safety management’ was added into the second survey questionnaire as a competency dealing with disaster situations. Japan is an earthquake-prone country and disaster-related medical management is inevitable. Thus, MLS must accomplish medical testing tasks under disaster situations like a medical professional, and it is necessary to prepare responses for all contingencies in nonemergency periods [34]. As MLSs, the novices were recommended to understand the preparation required in developing competencies.

The survey results showed that the main laboratory skill competencies of novice MLSs in the ‘Preparation and analysis (general) and (specimen)’ categories were relatively higher than those in other categories based on the IFBLS’s core competence. The IFBLS’s core competence was the ability of the standard expected in MLS, and ours was a lower level than the novice; however, several competencies in the ‘Preparation and analysis (general) and (specimen)’ categories were slightly lower than or similar to those of the IFBLS’s core competence. Regardless of the novice, the above-mentioned laboratory skills were considered essential and a minimum requirement in MLSs by expert MLSs. In addition, they were expected to have a general responsibility as any worker would have in a clinical setting. The results showed high consensus rates across categories in the first round. By adding new items, the participants provided suggestions in the first round, which also had a high consensus rate in the second round.

However, there were two outstanding competency differences in the competency of this study and that of IFBLS, which were referred to construct the draft competency. ‘Interpersonal development skills’, such as interpersonal relationships with health professionals in other institutions as well as with vendors and community members in IFBLS competency, were not required in this study; only basic communication skills for adults working in healthcare institutions were required. In addition, our competency of ‘professional development’ consisted of two domains, i.e. ‘Academic activity’ and ‘self-improvement’, which were unique compared to the IFBLS competency, which mainly covered the research planning in those categories. With a focus on self-study to enhance themselves, our competency considered job performances more reliable than strengthening the research skills of individuals. Nonetheless, the participant comments revealed that developing an ability to acquire a high level of knowledge and skills through self-learning is required from the start of a new job. Therefore, we changed from the ‘Research ability’ category to the present category and two domains. Limited to target workers in healthcare institutions and novice MLSs in this study, competency revealed the particular requirements for them. This is a functional finding because most qualified MLSs work at these institutions in Japan.

Further research is needed to explore the tools of assessments through the development of a competency scale, thereby helping to clarify the gaps of ability and correlated factors, which is also linked to study interventions. Furthermore, a scale for the unique competencies could also help assess the efficacy of educational programmes for Japanese MLS.

### The panel

The demographics of the study participants were estimated to be a group of expert-level MLSs with a mean ± SD of 32.4 ± 6.0 years of experience. Although the response rate of the first survey was low (26.5%), that of the second survey was high (95 of 106 [89.6%]). Regarding the low response rate in round 1, there might be two possible reasons. First, there is a possibility that the Japanese MLSs were unfamiliar with survey studies. There have not been many MLS survey studies in Japan, and one survey study revealed that the response rate of MLSs was 18.0%, which was lower than that reported in the present study [30]. However, the response rate in the present study was 26.5%, indicating that the panel was interested in the present study and exhibited professionalism as MLS experts. Second, our study period coincided with the beginning of the COVID-19 pandemic in Japan, during which clinical settings were expected to be hectic. On the other hand, the high response rate in round 2 suggested that the participants showed a particular interest in in-service education. The 95 participants fulfilled the requirement of the Delphi method (which requires a high participation number) [15]. The Delphi technique provided evidence of content and face validity, and the validity was influenced by the number of experts in the sample and the level of expertise and agreement [16]. This finding was suitable for those conditions and it confirmed that competency had content and face validity. Thus, the mean ± SD number of MLSs in the participant departments was 34.9 ± 22.1, suggesting that the head of MLSs managed sufficient staff and had an in-service education. The mean ± SD number of beds in the facilities to which the participants belonged was 499.8 ± 204.3, approximately the same as the national average of 475.1 beds for facilities with more than 300 beds. The institutes for the participants were scattered all over the country. The participants in this study were professionally experienced head MLSs with a high level of awareness and interest in in-service education and who could educate their staff. Additionally, the results are free from bias because of the facility size and regional characteristics.

### Limitations

First, the competencies were limited to MLSs working in medical institutions, which was a new challenge for Japanese MLS. As the competencies required may differ according to another field of activity, such as public health laboratories, clarifying the competencies required in other institutions is essential.

Second, the number of participants was limited. We distributed the second questionnaire only to the participants who consented to participate in our study and answered the first questionnaire. Therefore, fewer people participated in round 2 than in round 1. The number of participants in round 2 would have been much lower if we did not get as many responses in round 1. However, we considered that the participants could be regarded as they were either interested in MLS education or eager to work on it. Consequently, our participants have purified the expert's in MLS education among experts in MLS.

Third, the participants were from clinical settings only, except for MLS educators at universities and colleges. Opinions from school and clinical educators for MLSs must be investigated. To summarise those opinions, MLS competencies should be widely utilised.

Finally, the target institutions were limited to medical institutions with more than 300 beds, as they have sufficient numbers of MLSs, thus being able to ensure a proper educational system. Conversely, there may be difficulties in educating new appointees in small medical institutions with less than 300 beds. For instance, MLSs may not be available to spend enough time and effort on novice education because of the smaller number of personnel. Moreover, the scope of work may be broader than that of a large hospital, and duties with responsibility may be assigned at a relatively early stage.

This study revealed a tendency of new appointees to be required to have the ability to perform a wide range of laboratory tasks that were required as part of their relatively general responsibilities. The study participants affirmed that the seniors and supervisors should mainly take up responsibilities during laboratory tasks, while the novices need primary responsibilities, such as the responsibility regarding being members of an organisation and medical professionals. In the future, clarifying the actual conditions of working in small medical institutions and the issues involved in training novices will be crucial.

### Strengths and challenges

First, we believe that feedback regarding competencies can contribute to the development of in-service education. For supervisors, the competencies in this study provide the advantage that they can teach efficiently without dropping essential concepts. For novices, the clarification of the required competencies makes it easier for them to set their own goals and improve their work motivation. Although this study focused on the novice period, competency during this period can lead to competencies, such as mid-career MLS and senior MLS competencies, and it is thus expected to develop an MLS career ladder. Given that the career ladder standardises and visualises the growth process of the required competencies [22, 23], it can be expected to develop systematic in-service education according to career levels.

Second, the novice competency can be utilised for MLS school education. The MHLW has been promoting the standardisation of education for MLSs [3], and it is thought to contribute to clinical practice guidelines. Additionally, this study is based on the core competencies of the IFBLS, and we believe that the inclusion of the competencies will pave the way for the development of world-class personnel.

Third, competencies could use research on MLS education. Compared with medical doctors, nurses and other medical professionals, MLSs have fewer education-based studies and information in Japan. This deviation may conceivably be because there are fewer people involved in research into the education of MLSs and because it is considered less important than others. In the future, MLS education will be enhanced by subsequent studies, which will help strengthen the identity and professionalism of MLSs. In this study, the Delphi method was utilised to identify the competencies as the minimum requirement for entry-level MLSs by the head of MLSs at a medical institution. In addition to the validity assessment of the draft questionnaire, a Delphi survey was conducted, which revealed that the number of experts, level of expertise, their comments and agreement were sufficient to indicate the content and face validity of our competencies [16, 35, 36]. The expert group was willing to participate in the surveys, meaning that we could gather the opinions of those who are eager for clinical education.

## Conclusions

In conclusion, two surveys led to a convergence of keen expert opinions with a high consensus rate, which was more than 97.9%. Fifty-four competencies were identified for eight categories. The competencies were broadly consistent with those in other countries; however, some were unique to Japanese MLSs because Japanese MLSs handled the tasks of different professionals in other countries. Thus, we clarified that the requirements were specific to novice MLSs in Japanese medical institutions as a qualifier and professional MLS member.

## Supplementary Information

**Additional file 1.** Outcomes of the round 1.

**Additional file 2.** Japanese novice medical laboratory scientists’ competency.

## Data Availability

The datasets used and/or analysed during the current study are available from the corresponding author on reasonable request.

## Abbreviations

CDC: Centers for Disease Control and Prevention
COVID-19: Coronavirus disease 2019
IFBLS: International Federation of Biomedical Laboratory Science
MHLW: Ministry of Health, Labour and Welfare
MLS: Medical laboratory scientist
PCR: Polymerase chain reaction
SD: Standard deviation
