# Supplementary material for: Competency for Japanese novice medical laboratory scientists: a Delphi method
Source: BMC Med Educ. 2022 Dec 16;22:875. doi: 10.1186/s12909-022-03878-7 (PMC9756718; doi:10.1186/s12909-022-03878-7)
Supplement: Supplementary file 1 — Additional file 1. Outcomes of the round 1. [file 12909_2022_3878_MOESM1_ESM.pdf]

Additional file 1

Outcomes of the round 1

			%				Median	Modification to the round 2
			important	somewhat important	somewhat unimportant	unimportant		
			1	2	3	4		
Sample collection	General	1	83.7	15.2	1.0	0.0	4	Not modified
	Venipuncture	2	89.4	10.5	0.0	0.0	4	Not modified
Preparation and analysis (general)	Pre-examination process	3	83.8	14.3	1.9	0.0	4	Not modified
		4	79.0	19.0	1.0	1.0	4	Not modified
	Process during the examination	5	77.1	21.9	1.0	0.0	4	Not modified
		6	72.4	27.6	0.0	0.0	4	Not modified
		7	77.1	21.9	1.0	0.0	4	Not modified
	Post-examination process	8	77.1	21.9	1.0	0.0	4	Not modified
		9	73.3	23.8	1.9	1.0	4	Not modified
	Report test results	10	61.9	34.3	2.9	1.0	4	Modified
		11	68.6	28.6	2.9	0.0	4	Modified
		12	72.4	26.7	0.0	1.0	4	Modified
	Management of document	13	54.3	41.0	3.8	1.0	4	Modified
		14	40.0	53.3	5.7	1.0	3	Modified
	Management of equipment and inventory	15	82.9	15.2	1.9	0.0	4	Not modified
								Added a new item
		16	73.3	23.8	2.9	0.0	4	Not modified
		17	62.9	31.4	5.7	0.0	4	Modified
		18	47.6	47.6	3.8	1.0	3	Modified
Preparation and analysis (specimens)	Pre-examination process	19	77.1	20.0	1.9	1.0	4	Not modified
		20	89.5	9.5	1.0	0.0	4	Not modified
		21	91.4	8.6	0.0	0.0	4	Not modified
	Post-examination process	22	72.4	26.7	1.0	0.0	4	Not modified
		23	76.2	21.0	2.9	0.0	4	Not modified
	Reagent control and management	24	77.1	21.9	1.0	0.0	4	Modified
		25	65.7	30.5	2.9	1.0	4	Generated

			%				Median	Modification to the round 2
			important	somewhat important	somewhat unimportant	unimportant		
			1	2	3	4		
Preparation and analysis (physiology)	Pre-examination process	26	85.7	11.4	1.9	1.0	4	Not modified
		27	94.3	4.8	1.0	0.0	4	Not modified
								Added a new item
								Added a new item
	Process during the examination	28	82.9	17.1	0.0	0.0	4	Not modified
Medical safety management	Routine labour practices	29	47.6	41.9	9.5	1.0	3	Modified
		30	67.6	30.5	1.9	0.0	4	Modified
		31	82.9	17.1	0.0	0.0	4	Not modified
		32	88.6	11.4	0.0	0.0	4	Not modified
	Emergency response	33	66.7	32.4	1.0	0.0	4	Modified
		34	54.3	40.0	5.7	0.0	4	Modified
Interpersonal relationships	Cooperation with medical professionals	35	86.7	13.3	0.0	0.0	4	Not modified
		36	78.1	21.9	0.0	0.0	4	Modified
		37	59.0	38.1	2.9	0.0	4	Modified
								Added a new item
		38	43.8	48.6	7.6	0.0	3	Generated
		39	36.2	53.3	9.5	1.0	3	Modified
		40	60.0	39.0	1.0	0.0	4	Modified
Research ability	Academic activity	41	50.5	45.7	3.8	0.0	4	Modified
		42	56.2	40.0	3.8	0.0	4	Generated
		43	66.7	30.5	2.9	0.0	4	Modified
	Self-improvement	44	56.2	40.0	3.8	0.0	4	Modified
		45	73.3	23.8	2.9	0.0	4	Modified
								Added a new item
Ethics	Professional responsibility	46	87.6	11.4	0.0	1.0	4	Modified
		47	58.1	37.1	2.9	1.9	4	Modified
		48	72.4	25.7	1.9	0.0	4	Modified
								Added a new item
	Medical ethics	49	88.6	11.4	0.0	0.0	4	Modified
		50	60.0	38.1	1.9	0.0	4	Modified
		51	80.0	18.1	1.9	0.0	4	Not modified
