# Supplementary material for: Competency for Japanese novice medical laboratory scientists: a Delphi method
Source: BMC Med Educ. 2022 Dec 16;22:875. doi: 10.1186/s12909-022-03878-7 (PMC9756718; doi:10.1186/s12909-022-03878-7)
Supplement: Supplementary file 2 — Additional file 2. Japanese novice medical laboratory scientists’ competency. [file 12909_2022_3878_MOESM2_ESM.pdf]

Additional file 2

Japanese novice medical laboratory scientists' competency

Category	Domain		Competency item
Sample collection	General	1	Collects specimen from patients safely and appropriately
	Venipuncture	2	Performs safe and comfortable venous blood collection of patients and stores it in an appropriate container
Preparation and analysis (general)	Pre-examination process	3	Receives specimens appropriately
		4	Prepares necessary equipment and supplies for analysis
	Process during the examination	5	Prioritises analyses based on urgency and work efficiency
		6	Performs analyses of patients' specimens, utilising appropriate testing methods and techniques
		7	Ensures that the test results are based on appropriate quality control
	Post-examination process	8	Assesses the validity of test results based on the clinical patient information and previous results
		9	Considers retesting, additional tests and so forth as necessary within established guidelines
	Report test results	10	Interprets verified results and reports test results with evidence

		11	Reports critical test results in a way that draws attention to them, if guidance is provided
		12	Uses laboratory system appropriately for retesting and reporting test results
	Management of document	13	Stores and disposes test results and reports in consideration of personal information protection
		14	Understands manuals and protocols correctly
	Management of equipment and inventory	15	Manages the internal quality control of the test equipment being used
		16	Understands the assessment results based on the correct knowledge of external quality control, if guidance is provided
		17	Maintains the test equipment being used
		18	Notices and reports abnormalities and defects of the test equipment being used
		19	Coordinates the inventory of supplies and test-related items (e.g. reagents), if guidance is provided
Preparation and analysis (specimens)	Pre-examination process	20	Transports patients' specimens safely and appropriately
		21	Evaluates whether specimens appropriate for testing have been collected (confirmation of specimen types)

		22	Evaluates specimens' suitability before testing
	Post-examination process	23	Evaluates the validity of test results by specimen types
		24	Stores specimens safely and properly
	Reagent control and management	25	Prepares and adjusts reagents and solutions required for testing and manages them under the direction of a supervisor (including staining solutions and culture media)
Preparation and analysis (physiology)	Pre-examination process	26	Guides the patient (examinee) appropriately into the physiological laboratory
		27	Identifies the patient (examinee) properly prior to the physiological examination
		28	Explains the test to the patient (examinee) properly before the physiological examination
		29	Manages the safety control of physiological examination equipment
	Process during the examination	30	Performs safe and comfortable physiological examination of the patient
Medical safety management	Routine labour practices	31	Understands the safe and appropriate environment of a laboratory
		32	Identifies unsafe work practices and regulatory violations and consults with supervisors and senior staff as needed

		33	Understands various types of biohazards and follows correct procedures for specimen transport and disposal
		34	Possesses the correct knowledge of infection prevention measures and practices them
	Emergency response	35	Understands how to respond to emergencies, such as disasters and power outages, and is always prepared
		36	Follows the hospital's emergency response procedure manuals in the event of an acute deterioration of a patient (examinee)
Interpersonal relationships	Communication	37	Reports, communicates and consults on work-related matters
		38	Treats patients (examinees) with respect
		39	Communicates appropriately with medical professionals and related parties (including clinical trainees)
	Cooperation with medical professionals	40	Performs the role of a member of the laboratory, if guidance and support are provided
		41	Understands each role of medical professionals and collaborates with them, if guidance is provided

		42	Understands the importance of team medicine and participates as a member of the team, if guidance is provided
Professional development	Academic activity	43	Understands the importance of evidence-based medicine, if guidance is provided
		44	Participates in workshops and conferences to maintain and update scientific knowledge and skills
	Self-improvement	45	Identifies one's own issues and sets personal goals, if guidance is provided
		46	Reflects on personal growth and makes connections to the next stage of growth
		47	Recognises the importance of lifelong self-directed learning as a medical professional
Ethics	Professional responsibility	48	Reports one's actions/inactions (oversights) and prevents them from recurring
		49	Expresses one's own professional thoughts according to the situation
		50	Is aware of being a member of an organisation and acts responsibly in the tasks assigned
		51	Understands the principle of the medical institution and the goals of the laboratory one works for

Medical ethics	52	Is aware of professionalism and adheres to the profession’s code of ethics
	53	Understands basic laws and policies related to healthcare (e.g. Personal Information Protection Law, medical insurance system, etc.)
	54	Conducts tests (examinations) without disadvantages to the patient (examinee)
